# Mechanism of hysteresis for composite multi-halide and its superior performance for low grade energy recovery

**DOI:** 10.1038/s41598-018-38237-4

**Published:** 2019-02-07

**Authors:** Guoliang An, Liwei Wang, Jiao Gao, Ruzhu Wang

**Affiliations:** Institute of Refrigeration and Cryogenics, Key Laboratory of Power Machinery and Engineering of MOE, Shanghai Jiao Tong University, Shanghai, 200240 China

## Abstract

Sorption hysteresis commonly exists for different sorbents and has a great impact on the performance, and recently it was found that the multi-halide sorbents could reduce the hysteresis phenomena. Here we report the mechanism of the sorption hysteresis for multi-halide under equilibrium/non-equilibrium conditions and its superior performance for low grade energy recovery. We find that the inner reaction among different halides does not happen and contribute to sorption hysteresis in sorption/desorption phases under equilibrium conditions. While under non-equilibrium conditions, multi-halide sorbents reduce the hysteresis significantly (the average hysteresis temperature difference decreases from 23.4 °C to 7.8 °C at 4.41 bar). The phenomena is studied, and results show that the continuous reaction within different halides under heterothermic condition leads to an operable multi-stage reaction property, which corresponds to better flexibility and faster response to heat source. The utilization of solar energy as heat source for a cloudy day is analyzed, and multi-halide sorbent has much larger average refrigeration power (improved by 43%) and could work efficiently most of the time. Such characteristics are also prospective for other thermochemical reaction technologies, such as de-NOx and energy storage because of lower energy input and higher energy output features.

## Introduction

Solid sorption systems, which can be powered by solar energy^1–4^ or low-grade waste heat^5–8^ and utilize working pairs with zero ODP and GWP, have received continuous increased attention for refrigeration^9,10^, heat pump^11,12^, energy storage^13,14^, gas capture^15–17^ and elimination of NO_X_ emission^18^. For solid chemisorption, both the sorption and desorption reaction need the driving force of chemical potential. For the sorption reaction, the temperature of the sorbents has to be lower than the threshold temperature and for the desorption reaction the opposite condition needs to be satisfied. For this reason, the sorption hysteresis phenomenon is a nature property, ubiquitously exists and has different effect for various applications. For example, the feature of larger hysteresis is preferred to make the capture be more completed under the condition of gas separations^19–21^, while the situation is opposite for thermal energy storage or refrigeration, for which larger hysteresis leads to much higher desorption temperature as well as the apparent reduction of exergy efficiency^22,23^. As a typical kind of solid chemisorption working pair for refrigeration, energy storage and NO_x_ elimination, halide-ammonia has been studied extensively^24–28^. For such type of sorbents the obvious sorption hysteresis commonly exists^29,30^ and could not be neglected. Recently it was found that compact composite multi-halide sorbents (NH_4_Cl/CaCl_2_/MnCl_2_-NH_3_)^31^ could reduce the sorption hysteresis significantly. The preliminary prediction thought that it might be caused by the resorption among different halides, but it was not investigated with detailed analysis and experiments.

In this work, we aim to reveal the mechanism for hysteresis of multi-halide. Because the practical hysteresis of multi-halide relies on both chemical reaction and heat/mass transfer, it is necessary to carry on equilibrium experiments first to research the hysteresis of complexation reaction between solid sorbent and sorbate. After that, non-equilibrium experiments are proceeded to analyze the complexation reaction under the impact of heat and mass transfer. Under equilibrium conditions, for each reaction point, temperature will not be changed until sorption/desorption phase reaches equilibrium. First because the mass of solid sorbent is less than 0.2 g, and meanwhile the testing process for each cycle can last over 3 days, the influence of heat and mass transfer on testing results can be neglected. Under non-equilibrium conditions, temperature of thermostatic bath is increased from ambient temperature to maximum desorption temperature (or decreased from maximum desorption temperature to ambient temperature) within one hour without steady intermediate processes, meanwhile the mass of the solid sorbent is over 200 g, thus, heat and mass transfer will influence the sorption performance dramatically.

Results show that for multi-halide the resorption does not happen. The discovery suggests that the combination of the multi-stage reaction and non-equilibrium heat and mass transfer leads to a better flexibility and faster response to heat source, corresponding to the minor hysteresis phenomena. Such a result also leads to a prospective performance for real application of energy conversion and utilization.

## Solid Sorption and Resorption Processes

**Figure 1:**
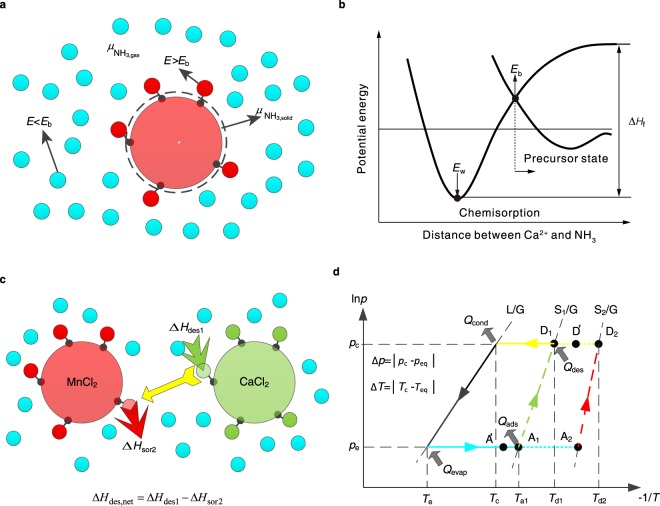
Solid chemisorption and resorption processes. (**a**) Solid chemisorption schematic diagram, showing the role of chemical potential and activation energy. (**b**) The precursor state of chemisorption, using CaCl_2_-NH_3_ as example. (**c**) Resorption hypothesis inside multi-halide sorbent, using CaCl_2_ and MnCl_2_ as example. (**d**) The Clapeyron figure of solid chemisorption. S represents solid halide, and G refers to the reaction gas.

The analysis of Gibbs free energy is widely applied in physical adsorption to research equilibrium adsorption property. Both Dubinin-Polanyi potential energy theory and Dubinin-Radushkevish theory are based on analysis of Gibbs free energy and adsorption potential, which are essential to study physical adsorption with various materials and bore diameter. However, for chemisorption such as reaction between halide and ammonia, analysing Gibbs free energy can only obtain the reaction direction but not the specific sorption property, as the sorption performance is led by the complexation reaction^32^. The principle of single-stage solid sorption cycle, taking MnCl_2_ as example, can be divided into two phases, i.e. sorption phase and desorption phase (shown in Supplementary Fig. 1). In the desorption phase, heat from solar energy or industry is transferred into [Mn(NH_3_)_6_]Cl_2_ for completing desorption process and transferring sorbate vapour into ammonia source (working as condenser). In the sorption phase ammonia will evaporate and be sorbed by [Mn(NH_3_)_2_]Cl_2_, in which the heat released could be supplied to user side. Two necessary conditions need to be satisfied for chemisorption reaction between halide-ammonia working pairs, as shown in Fig. 1a,b and Equation (1). Considering the reaction direction of chemisorption related with Gibbs free energy, the first condition (Fig. 1a) is that the chemical potential of ammonia in the gas phase (${{\mu }}_{{{\rm{NH}}}_{3,{\rm{gas}}}}$) is larger than that of ammonia equilibrated with the solid phase^33^ (${{\mu }}_{{{\rm{NH}}}_{3,{\rm{solid}}}}$), i.e. in Equation (1) the Δ*μ* is larger than 0. Meanwhile, the sorption force is huge enough for the reaction point to overcome the precursor state (potential energy barrier, *E*_b_) and come into the potential well (*E*_w_) of chemical reaction^34^ (Fig. 1b). Such two conditions are required for both single halides and multi-halide sorbents.

1 $${\rm{\Delta }}\mu ={\mu }_{{{\rm{NH}}}_{3,{\rm{gas}}}}-{\mu }_{{{\rm{NH}}}_{3,{\rm{solid}}}}=RT\,\mathrm{ln}\,(\frac{{p}_{{{\rm{NH}}}_{3,{\rm{gas}}}}}{{p}_{{{\rm{NH}}}_{3,{\rm{solid}}}}})$$

where Δ*μ* is the chemical potential difference between gas source and solid-gas interface (J·mol^−1^), *R* is ideal gas constant (J·mol^−1^·K^−1^), *T* is the temperature of gas source (K), ${p}_{{{\rm{NH}}}_{3,{\rm{gas}}}}$ and ${p}_{{{\rm{NH}}}_{3,{\rm{solid}}}}$ are pressure of gas source and solid-gas interface respectively (Pa).

When two or more halides are complexed, the situation may become complicated because of the possible interaction among different halides. Take MnCl_2_ and CaCl_2_ as example, and the analysis is shown in Fig. 1c,d. In Fig. 1d, ‘D’ represents desorption while ‘A’ stands for sorption. When the state of halide-gas interface is *D*’ which is between S_1_/G and S_2_/G line (Fig. 1d), S_2_ has ability to sorb while S_1_ desorbs. Such as CaCl_2_ and MnCl_2_, for which the positions in Fig. 1d are corresponding to S_1_ and S_2_, respectively, the resorption process (shown in Fig. 1c) might happen between sorbents instead of gas source. In this case, S_1_ will sorb heat and S_2_ will release heat simultaneously. Thus, the sum of enthalpy for S_1_ to desorb will be reduced, as shown in Fig. 1c. As the activated energy of desorption is the sum of the desorption enthalpy and the activated energy of sorption, the activated energy of desorption is much easier to be overcome for the reaction between two types of halides, which might be beneficial to reduce desorption hysteresis. In order to figure out if such a resorption process exits, composite sorbents with porous thermal conductive matrix are developed (see Supplementary Fig. 2 for preparation procedures of composite sorbents), and series of equilibrium experiments are performed.

## Single Halide Sorbents Under Equilibrium Conditions

NH_4_Cl is a special halide, whose Clapeyron line is the closest to NH_3_ among all of the known halides. However, it cannot be applied alone in the reaction system due to the chemical instability under high temperature ((see Supplementary Fig. 3a for the pre-treatment result of NH_4_Cl). Because of the decomposition of NH_4_Cl, only CaCl_2_ and MnCl_2_ are tested as single halide sorbents by Rubotherm balance (see Supplementary Fig. 4a for schematic diagram of Rubotherm balance test unit) under equilibrium conditions. The threshold temperatures of sorption and desorption under various pressures are measured for the calculation of reaction enthalpy and entropy. During the measurement, the valve between ammonia vessel and measuring chamber is always open. For the desorption process, the initial temperature is near the ambient temperature. First, the thermal jacket is heated by the thermostatic bath till the temperature inside the measuring chamber is 10 °C lower than the theoretical threshold temperature. Second, the set temperature of thermostatic bath is increased by 5 °C by each step when temperature of measuring chamber in the previous step is stable. It is expected that the sudden change of mass sorption rate would happen under a certain temperature. That point is regarded as the practical threshold temperature of desorption. For the CaCl_2_-NH_3_ working pair, there are two reaction stages under the test conditions, as shown in Equation (2) and Equation (3), thus two values of threshold temperature should be obtained during the desorption process.

2 $$[{\rm{Ca}}{({{\rm{NH}}}_{3})}_{8}]{{\rm{Cl}}}_{2}\iff [{\rm{Ca}}{({{\rm{NH}}}_{3})}_{4}]{{\rm{Cl}}}_{2}+4{{\rm{NH}}}_{3}$$

3 $$[{\rm{Ca}}{({{\rm{NH}}}_{3})}_{4}]{{\rm{Cl}}}_{2}\iff [{\rm{Ca}}{({{\rm{NH}}}_{3})}_{2}]{{\rm{Cl}}}_{2}+2{{\rm{NH}}}_{3}$$

While for MnCl_2_-NH_3_ working pair, only one reaction stage exits:

4 $$[{\rm{Mn}}{({{\rm{NH}}}_{3})}_{6}]{{\rm{Cl}}}_{2}\iff [{\rm{Mn}}{({{\rm{NH}}}_{3})}_{2}]{{\rm{Cl}}}_{2}+4{{\rm{NH}}}_{3}$$

These three reaction stages are simplified as Ca 8-4, Ca 4-2 and Mn 6-2 respectively. For the sorption process, the measurement procedures are similar but the initial desorption temperature should be high enough.

**Figure 2:**
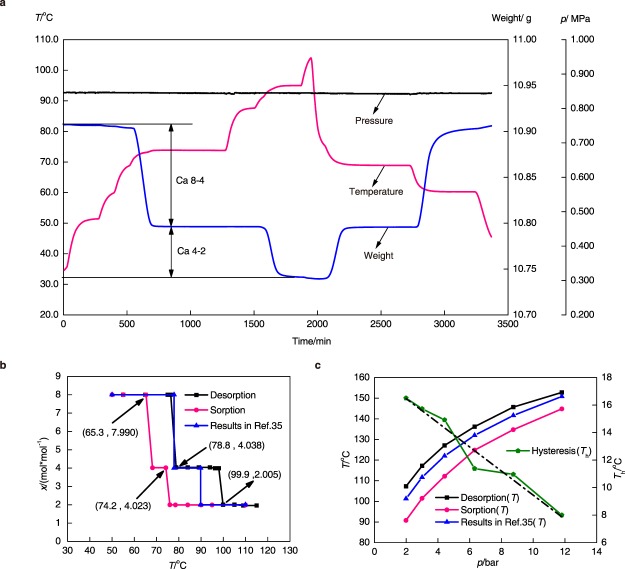
Experimental results of single halide sorbents under equilibrium conditions. (**a**) Isobaric sorption/desorption processes of CaCl_2_-NH_3_ working pair tested by Rubotherm balance. (**b**) The typical sorption/desorption hysteresis phenomenon under 0.851 MPa of CaCl_2_-NH_3_ working pair. (**c**) Comparison among sorption, desorption, and the value in ref.^34^ from 0.199 MPa to 1.172 MPa for MnCl_2_-NH_3_ working pair.

The typical testing process is shown in Fig. 2a, using CaCl_2_-NH_3_ working pair under 0.851 MPa as example. The pressure of system can keep stable during the whole test process, while with temperature rising or decreasing the weight measured by the magnetic balance changes, i.e. obvious reaction stages can be obtained. The results of threshold temperature of sorption and desorption processes for CaCl_2_ under 0.851 MPa are shown in Fig. 2b (expressed by sorption quantity, *x*). It can be seen that the difference between sorption and desorption processes of CaCl_2_-NH_3_ is obviously existed and the sorption hysteresis is large, which means higher heating temperature is required for desorption phase, comparing the data without hysteresis in reference^35^. For MnCl_2_-NH_3_ only one reaction stage exits and all of the test maximum cycle sorption quantities are extremely close to 4 mol·mol^−1^ under various conditions. The corresponding relations between pressure (from 0.199 MPa to 1.172 MPa) and threshold temperature are shown in Fig. 2c, in which the hysteresis is calculated by the temperature difference between desorption and sorption temperature. The hysteresis decreases when the pressure increases, which indicates increasing the chemical potential of gas source can decline the hysteresis.

## Multi-Halide Sorbents Under Equilibrium Conditions

Differing from NH_4_Cl, the multi-halide sorbent (NH_4_Cl & CaCl_2_ & MnCl_2_) keeps steady state even though the heating temperature is up to 180 °C (see Supplementary Fig. 3b for the pre-treatment result of multi-halide sorbent). For multi-halide sorbents, the proportion of different halides needs to be determined before the test. CaCl_2_ has the largest cycle sorption quantity (6 mol ammonia/mol halide), so the reduction of its hysteresis is the key point for improving the cycle performance of sorbent. If the resorption happens for same cycle mass of sorbate among different halides (NH_4_ X-0 with Ca 8-4, and Ca 4-2 with Mn 6-2), the mass of different sorbents should be matched each other. However the sorption process of NH_4_Cl is bivariate controlled, which means the maximum molar cycle sorption quantity of it is not constant as CaCl_2_ and MnCl_2_. Therefore, the proportion is designed based on the relative molecular mass and molar cycle sorption quantity of CaCl_2_ and MnCl_2_ (3:1.7) and the mass proportion of NH_4_Cl:CaCl_2_:MnCl_2_ is chosen as 1:3:1.7.

**Figure 3:**
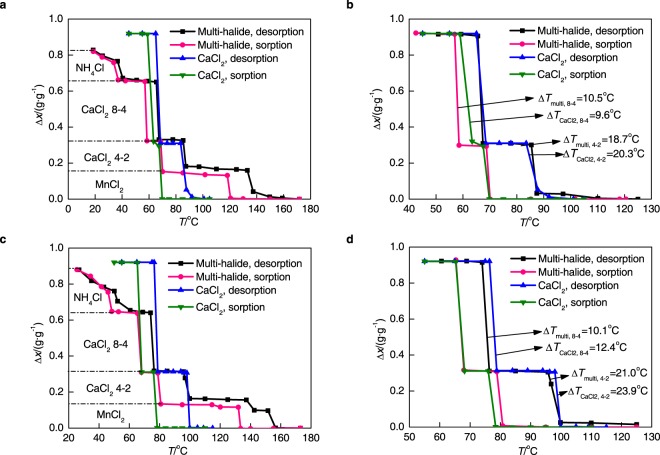
Comparison between multi-halide sorbent (1:3:1.7) and CaCl_2_ for isobaric sorption/desorption curves and hysteresis phenomenon. (**a**,**c**) For multi-halide sorbent, the sorption quantity is the sorbed mass of ammonia by multi-halide sorbent over the mass of multi-halide sorbent itself, at 0.622 MPa (**a**) and 0.851 MPa (**c**) respectively. (**b**,**d**) For the CaCl_2_ inside multi-halide sorbent, the sorption quantity is the sorbed mass of ammonia by CaCl_2_ over the mass of CaCl_2_ itself, at 0.622 MPa (**b**) and 0.851 MPa (**d**) respectively.

Under the similar operation conditions with that for single halide sorbents, isobaric sorption/desorption curves of NH_4_Cl/CaCl_2_/MnCl_2_-NH_3_ working pair under two typical working conditions are shown in Fig. 3 (expressed by cycle sorption quantity, Δ*x*), taking the hysteresis of CaCl_2_ as a criterion. Figure 3a,c show that the hysteresis area of multi-halide sorbent is smaller than that of CaCl_2_, if considering the temperature range lower than 100 °C, which is coincident with the results of our previous study^24^. The hysteresis area means cycle sorption quantity (Δ*x*) multiplies hysteresis temperature difference (Δ*T*). There are two reasons for that why hysteresis area is used. First is that before dividing reaction of CaCl_2_ from multi-stage reaction (Fig. 3a,c), it is difficult to compare hysteresis temperature difference directly between multi-halide and single halide (CaCl_2_) because they are multi-stage reaction and single-stage reaction respectively. Second, for sorption/desorption processes both sorption quantity (vertical axis) and the temperature (horizontal axis) are all important parameters, and the area can reflect these two parameters with one index. The reason of hysteresis area decrease is just because the combination of multi-stage reaction makes hysteresis area smaller than that of single halide (CaCl_2_) mathematically. But to figure out the impact of mixing halides on the chemical reaction hysteresis of CaCl_2_, the sorption quantity belongs to CaCl_2_ inside multi-halide sorbent is selected and compared with single halide of CaCl_2_. The comparison sorption quantity should be the sorbed mass of ammonia by CaCl_2_ over the mass of CaCl_2_ itself for both multi-halide sorbent and CaCl_2_, i.e. $x={m}_{{{\rm{NH}}}_{3}}/{m}_{{{\rm{cacl}}}_{2}}$. The results are shown in Fig. 3b,d respectively. Under equilibrium condition, the reaction stage is clear to be distinguished, and the hysteresis temperature difference is defined to describe the hysteresis characteristic. If the resorption process occurred, the reaction hysteresis of CaCl_2_ in multi-halide sorbent should be different from the single halide of CaCl_2_-NH_3_ working pair. The comparison results show that the hysteresis of reaction Ca 8-4 only slightly increases (from 9.6 °C to 10.5 °C in Fig. 3b) while that of Ca 4-2 only slightly reduces (from 20.3 °C to 18.7 °C in Fig. 3b). Considering the maximum absolute error of hysteresis temperature difference is about 3 °C due to the operation and calculation method, the whole influence on Ca 8-2 is so weak that the difference can be neglected. Thus it is deduced that the resorption does not occur. Different halides react independently without additional reactions means the activated energy will not be changed. The equilibrium performance of multi-halide sorbent does not differ from the combination of sorption/desorption effects of single halide sorbents much.

## Experimental Results Under Non-Equilibrium Conditions

**Figure 4:**
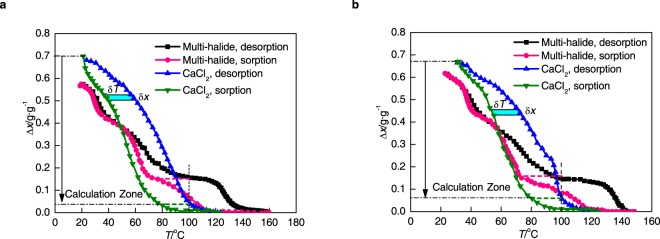
Comparison between multi-halide sorbent (1:1:1) and CaCl_2_ under non-equilibrium conditions. (**a**,**b**) Non-equilibrium sorption/desorption curves and hysteresis phenomenon at 0.441 MPa (**a**) and 0.622 MPa (**b**) respectively.

Under equilibrium conditions, sorption hysteresis has nothing to do with heat and mass transfer, as it is only influenced by chemical reaction over potentials. The non-equilibrium condition is defined as the condition impacted by heat and mass transfer, which is typical for sorption refrigeration, heat pump, energy storage (thermal cell) or de-NOx processes. It has been proved that resorption does not occur in equilibrium experiments. In the following non-equilibrium experiments, there is still not resorption because only heat and mass transfer effect would not lead to new inner reaction. In order to investigate the mechanism of hysteresis under non-equilibrium conditions, the test unit with PDM (pressure differential meter) is used. The sorption bed is heated or cooled with full power, making the temperature change from 35 °C to 160 °C without steady intermediate processes. The experimental results can be seen in Fig. 4a,b (expressed by cycle sorption quantity, Δ*x*). The interesting phenomenon is that the hysteresis area of multi-halide sorbent is much smaller than that of CaCl_2_ within temperature lower than 100 °C, for which the desorption process of CaCl_2_ could almost complete thoroughly. Because under non-equilibrium condition, the reaction stage is not obvious enough, the hysteresis temperature difference cannot be obtained directly. Thus, the average hysteresis temperature difference (*T*_hys_) is defined, which can also qualitatively calculate the irreversible loss caused by hysteresis:

5 $${T}_{{\rm{hys}}}=\frac{\{\sum _{i=1}^{{{\rm{N}}}_{{\rm{d}}}-1}[({x}_{{{\rm{d}}}_{i}}-{x}_{{{\rm{d}}}_{i+1}})\times {T}_{{{\rm{d}}}_{i+1}}]-\sum _{i=1}^{{{\rm{N}}}_{{\rm{s}}}-1}[({x}_{{{\rm{s}}}_{{{\rm{N}}}_{{\rm{s}}}-i+1}}-{x}_{{{\rm{s}}}_{{{\rm{N}}}_{{\rm{s}}}-i}})\times {T}_{{{\rm{s}}}_{{{\rm{N}}}_{{\rm{s}}}-i+1}}]\}}{{x}_{{{\rm{d}}}_{1}}-{x}_{{{\rm{d}}}_{{{\rm{N}}}_{{\rm{d}}}}}}$$

where *x* is the sorption quantity (g·g^−1^), superscript ‘d’ represents data of desorption, while superscript ‘s’ stands for data of sorption.

**Figure 5:**
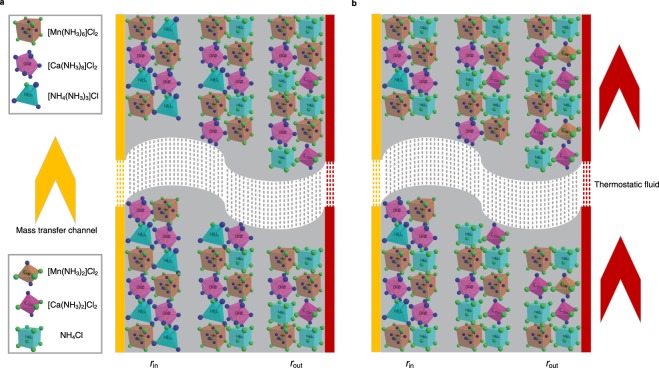
Relationship between spatial position of sorption bed (*r*) and reaction state of different halides inside multi-halide sorbent, during whole desorption stages under non-equilibrium conditions. (**a**) Temperature reaches desorption temperature of CaCl_2_ and two halides (NH_4_Cl and CaCl_2_) react simultaneously. (**b**) Temperature reaches desorption temperature of MnCl_2_ and two halides (CaCl_2_ and MnCl_2_) react simultaneously.

Figure 4a shows that when the evaporation pressure is controlled at 0.441 MPa, *T*_hys_ of CaCl_2_ is 23.4 °C, while that of multi-halide sorbent is dramatically reduced by 2/3, to 7.8 °C. Similar result is obtained under 0.622 MPa (Fig. 4b), from 21.2 °C to 7.2 °C. The reason for such minor hysteresis phenomena of multi-halide sorbent is analysed. Under equilibrium condition, the sorption/desorption process occurs in order (Fig. 3a,c) because it is only related with chemical reaction. However, under non-equilibrium condition, because of the synthetic effect of rapid temperature increase and big temperature difference among various positions, the simultaneous reaction process of different halide exits, as shown qualitatively in Fig. 5.

Taking desorption process as example, due to the existence of heat transfer temperature difference, the temperature of the material near to external *r*_out_ is higher than that near to inner *r*_in_, which causes the reaction rate of sorbent in external layer larger than that in the inner layer. Thus, when temperature is just over threshold temperature of NH_4_Cl, only part of [NH_4_(NH_3_)_3_]Cl located at *r*_out_ begins to desorb. While during stage in Fig. 5a, before all of the NH_4_Cl sorbents complete desorption process, [Ca(NH_3_)_8_]Cl_2_ becomes active to desorb. This duration is called simultaneous reaction stage. The similar process (Fig. 5b) exists between middle and high temperature halide sorbents, which means better flexibility and faster response to heterothermic condition due to the multi-stage reaction property. Meanwhile, mass transfer through the reaction medium (composite sorbent) need to be considered under two dimensions. The grain is the basic unit where the reaction takes place, while the pellet is a combination of reactive grains. Mass transfer contributes to the difference between test under equilibrium conditions and non-equilibrium conditions, and bad mass transfer will enlarge sorption hysteresis under non-equilibrium conditions. However, for halide-ammonia working pair the pressure is generally high, so the mass transfer performance is commonly acceptable, and the performance is mainly limited by the heat transfer. Thus, mass transfer effect is not discussed in detail for this part.

The reason that multi-stage reaction could decrease the practical hysteresis under non-equilibrium conditions is that it makes different halides react simultaneously under various reaction positions in the reactor. As no resorption process occurs, the sorption/desorption temperature of CaCl_2_ molecule is not changed. However, the simultaneous multi-stage reaction under non-equilibrium conditions makes average desorption temperature of CaCl_2_ inside multi-halide lower than single halide of CaCl_2_ with the same sorption quantity (due to desorption of NH_4_Cl in advance) while average sorption temperature of CaCl_2_ higher (due to sorption of MnCl_2_ in advance). Thus, the continuous reaction process combined with non-equilibrium heat and mass transfer leads to higher sorption temperature and lower desorption temperature of multi-halide sorbent than that of single halide (CaCl_2_) under the same condition, and such a phenomenon reduce the sorption hysteresis effectively and could adapt well to the solar energy.

## Performance of Refrigeration

**Figure 6:**
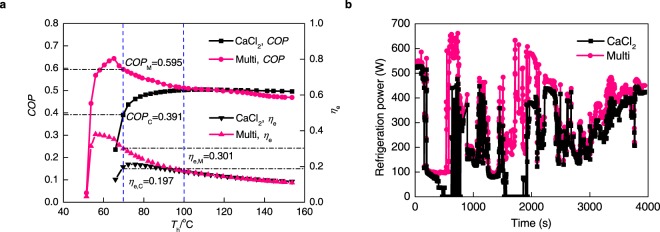
Non-equilibrium conditions for multi-halide sorbent and CaCl_2_. (**a**) Energy and exergy efficiency under non-equilibrium conditions for multi-halide sorbent and CaCl_2_, data from test unit with PDM. The uncertainty of the calculated data is shown in Fig. 8. (**b**) Refrigeration power on cloudy day, with solar energy as heat source, using multi-halide sorbent and CaCl_2_.

To prove the superior property of minor hysteresis phenomena of multi-halide sorbent under non-equilibrium conditions, the performance of the basic refrigeration cycle is analysed. The refrigeration coefficient of performance (*COP*) which is calculated by ignoring the thermal capacity of the liquid refrigerant and bed metal, is defined as:

6 $$COP=\frac{{Q}_{{\rm{ref}}}}{{Q}_{{\rm{des}}}}=\frac{{Q}_{{\rm{ref}}}}{{Q}_{{\rm{s}}}+{\rm{\Delta }}{H}_{{\rm{d}}}}=\frac{L\sum {\rm{\Delta }}{x}_{i}}{\overline{{c}_{{\rm{halide}}}}({T}_{{\rm{h}}}-{T}_{1})+\sum {\rm{\Delta }}{x}_{i}{h}_{di}}$$

where *L* is latent heat of vaporization of ammonia (J∙kg^−1^), ∆*x*_*i*_ is cycle sorption quantity (kg∙kg^−1^) of various halide sorbents,$\bar{{c}_{{\rm{h}}{\rm{a}}{\rm{l}}{\rm{i}}{\rm{d}}{\rm{e}}}}$ is the average specific heat of the multi-halide sorbent (J∙kg^−1^∙K^−1^), *T*_h_ is the temperature of the heat source, *T*_1_ is the cold temperature of the sorption bed (10 °C higher than the ambient temperature) and *h*_*di*_ is the desorption heat (J∙kg^−1^). The reaction heat is obtained from Supplementary Table 1. The exergy efficiency of the sorption refrigeration cycle is:

7 $${\eta }_{{\rm{e}}}=\frac{{Q}_{ref}(\frac{{T}_{0}}{{T}_{e}}-1)}{({Q}_{s}+{Q}_{d})(1-\frac{{T}_{0}}{{T}_{{\rm{h}}}})}=COP\cdot \frac{\frac{{T}_{0}}{{T}_{e}}-1}{1-\frac{{T}_{0}}{{T}_{{\rm{h}}}}}$$

where *T*_e_ is the evaporation temperature (0 °C), *T*_0_ is the environment temperature (20 °C). The performance of CaCl_2_ and multi-halide sorbent under non-equilibrium conditions is shown in Fig. 6a. The uncertainty of the calculated *COP* and ${\eta }_{{\rm{e}}}$ are shown in Supplementary Fig. 8.

Figure 6a shows that in CaCl_2_ reaction zone (with heat source temperature between 70 °C and 100 °C), due to reduction of hysteresis multi-halide sorbent has great advantages over CaCl_2_. Hysteresis decrease means the driven desorption temperature (*T*_h_) could be decreased, thus COP shown in Equation (6) will increase. If the heat source temperature is lower than 65 °C, the cycle sorption quantity for single halide of CaCl_2_ is 0, while for multi-halide sorbent, the system performance could be at a steady high level even though the heat source temperature is around 50~65 °C because of the existence of NH_4_Cl, which has small reaction enthalpy that will be helpful for the improvement of the COP. It need be mentioned that because the desorption and sorption phases switch intermittently during the whole cycle, the adsorber-heat exchanger configuration will affect sensible heat (Qs in Equation (6)) obviously, and then will influence COP. Thus, for the application of working pairs in sorption systems, generally for calculating COP, the sensible heat of sorption bed should be considered and the data will be around 10–25%^36^ lower than that in Fig. 6a.

As we have not done experiments utilizing solar energy, we indirectly analysis the test results of absorber temperature and solar flux from reference^37^. Because unsteady conditions need to be paid more attention to ensure the performance stability of materials, we choose data under cloudy day. The calculation steps of refrigeration power are as follows: According to absorber temperature versus time, COP versus time could be obtained with data in Fig. 6a. After that the initial data of solar flux and sorption bed temperature are gotten from ref.^37^, which means *Q*_des_ in Equation (6) is given. Then the refrigeration power can be calculated by Equation (8):

8 $${\rm{Refrigeration}}\,{\rm{Power}}=COP\times {\rm{Solar}}\,{\rm{Flux}}\times \delta $$

where $\delta$ is 1 when the sorption bed temperature is higher than threshold desorption temperature (*T*_h_), i.e., desorption process can proceed; otherwise $\delta$ equals to 0.

The refrigeration power (per unit area) by utilizing multi-halide sorbent and CaCl_2_ are analysed, and the results are shown in Fig. 6b. The average refrigeration power of multi-halide sorbent is 321 W, while that of CaCl_2_ is just 224 W. Meanwhile, the refrigeration system with CaCl_2_ will become invalid during 16% of the cloudy day because of the low heating temperature from solar energy, but multi-halide sorbent could solve this problem successfully.

As mentioned previously, although the NH_4_Cl has excellent theoretical sorption property for low grade energy recovery, it cannot be used in the system alone because of its decomposition phenomena. By using multi-halide sorbent, the chemical stability of NH_4_Cl can be obtained, which makes the multi-halide sorbent has a high efficiency in wider temperature range.

## Conclusions

In summary, the hysteresis mechanism of multi-halide sorbent under both equilibrium and non-equilibrium conditions are investigated. The hysteresis characteristics of CaCl_2_ and multi-halide sorbents are compared, and the results show that under equilibrium condition the hysteresis characteristic of CaCl_2_ does not change inside the multi-halide sorbent, which means the inner resorption does not occur. The non-equilibrium experiments are carried out, including the influence of chemical reaction, heat transfer, and mass transfer. Results show that the multi-halide sorbent could decrease the hysteresis temperature difference significantly from 23.4 °C to 7.8 °C if compared with CaCl_2_ when the evaporation pressure is controlled at 0.441 MPa. The reason is analysed and it is caused by the better flexibility and faster response to heterothermic condition due to the multi-stage reaction property. The *COP* and ${\eta }_{e}\,$ of non-equilibrium condition for refrigeration are analyzed, and multi-halide sorbent adapts to low temperature heat source much better. The utilization of solar energy as heat source for a cloudy day is analyzed, and multi-halide sorbent has much larger average refrigeration power (improved by 43%) and could work efficiently most of the time. Therefore, multi-halide sorbent is prospective for low temperature heat source conditions and variable heat source conditions because of the operable multi-stage reaction property and less hysteresis.

Such a conclusion is also applicable for other thermal chemisorption processes, such as de-NOx and thermal energy storage processes. For de-NOx processes it may decrease the energy consumption significantly, and for energy storage processes the higher energy quality output combined with large energy density makes it a promising technology.

## Methods

### Preparation of composited sorbents used in Rubotherm balance

Previous studies show that expanded nature graphite treated by the sulphuric acid (ENG-TSA) could prevent agglomeration and improve the heat transfer performance significantly for halides due to its porous property and high conductivity^38,39^, so it is selected as the matrix of composite sorbents. The mass of sorbent used in the experiments is less than 0.2 g. First, different halides are mixed according to the proportion and pure water is added by dropper into the mixture to make them dissolve. Sitting the solution until sorbents are exactly dissolved, and then the beaker is put into oven and heated over 200 °C for 2 h to eliminate the crystal water inside halides. At last, the powder-like multi -halide sorbent is mixed with a layer of ENG-TSA.

### Preparation of composited sorbents used in test unit with PDM

For the test unit with PDM, the mass of sorbent is generally 180 g to 200 g. First, different halides are mixed according to the proportion and pure water is added to the mixture to make them dissolve. Sitting the solution until sorbents are dissolved. The ENG-TSA is mixed with the sorbent solution directly and then the mixture is put into the oven and heated over 200 °C for 8 h to eliminate the crystal water inside halides. Finally, the composite sorbents are compacted to density of 500 kg/m^3^.

### Measurement of sorption quantity by Robotherm balance

The Rubotherm balance is used for researching hysteresis mechanism under equilibrium conditions, for which the heat/mass transfer influence on sorption quantity can be neglected thoroughly because the mass of sorbents is less than 0.2 g. Supplementary Fig. 4a presents the schematic diagram of Rubotherm balance test unit. The overall device includes a Rubotherm balance, a thermostatic oil bath, a thermostatic glycol water bath, and a vacuum pump. The resolution of the balance is 10 μg. The typical sample mass is 0.1 g to 0.2 g of dry sorbent, which is put in a small steel basket whose weight is about 10 g. The basket is suspended inside the sealed steel chamber, whose temperature is controlled by a thermal jacket through thermal radiation via the circulation oil. The oil temperature is controlled by the thermostatic bath (Julabo SE-6) with ±0.01 K temperature accuracy. The measuring chamber is connected with an ammonia reservoir which acts as the evaporator/condenser, whose temperature is controlled by a thermostatic glycol water bath (Julabo F32-ME) with the temperature accuracy of ±0.01 K. The working pressure of measuring chamber is under the saturation pressure of the ammonia. A PT100 temperature sensor with an accuracy of 0.1 K is located beneath the basket to monitor the temperature of the sample, while the pressure of the vapour is detected by a calibrated absolute pressure sensor (Druck DPI 282) with the precision of 0.04%. It is difficult to compress the mixture without mass loss, which will cause big error because of the small data of mass. The density of the whole sorbent is lower than 1 g/cm^3^, so a round steel wire mesh is covered over the sorbents inside the basket to prevent the mass loss in evacuating and testing processes. Although the sorbent has been dried before the beaker is installed in the equipment, it still needs to be dried further at 180 °C by the operation of the thermostatic bath for several hours until the mass change is negligible. Then the system is evacuated to make sure that all the gas has been eliminated from the measuring chamber.

### Measurement of sorption quantity by test unit with PDM

The sorption quantity of various sorbents can be tested under equilibrium or non-equilibrium conditions by the test unit with PDM (see Supplementary Fig. 4b for the schematic diagram of test unit with PDM), for which composite sorbent is compacted inside as a block directly. The test unit consists mainly of a sorption bed, one refrigerant vessel served as evaporator or condenser, two cryostats, one pressure differential meter, etc. The sorbent mass is measured by the balance (BS2202S) with a measuring error of 0.01 g. The temperature is tested by the PT100 temperature sensor with an error of 0.15 K. The pressure and pressure difference are tested by the smart pressure transmitter and PDM, whose testing errors are 0.25% and 0.65% respectively. For testing the equilibrium isobaric sorption/desorption performances, for sorption process, open the valve between the sorption bed and evaporator. Keep the refrigerant vessel at a constant pressure by controlling the temperature of the cryostat connected with it. Meanwhile, decrease the temperature of sorbent bed from high temperature to ambient temperature by 5 °C each time. Until the data for sorption quantity does not change for 10 min at least, and then decrease the temperature to the next point. For desorption process, in a similar manner, sorption bed is heated and the data for sorption quantity and temperature are collected. For testing the non-equilibrium sorption/desorption performances, the sorption bed is heated or cooled with full power, making the temperature change from 25 °C to 160 °C without steady intermediate process.

## Supplementary information

Mechanism of hysteresis for composite multi-halide and its superior performance for low grade energy recovery

## Data Availability

The authors declare that the main data supporting the findings of this study are available within the article and its Supplementary Information files. Extra data are available from the corresponding author upon request.
