# Supplementary material for: Mechanism of hysteresis for composite multi-halide and its superior performance for low grade energy recovery
Source: Sci Rep. 2019 Feb 7;9:1563. doi: 10.1038/s41598-018-38237-4 (PMC6367323; doi:10.1038/s41598-018-38237-4)
Supplement: Supplementary file 1 — Mechanism of hysteresis for composite multi-halide and its superior performance for low grade energy recovery [file 41598_2018_38237_MOESM1_ESM.docx]

**Mechanism of hysteresis for composite multi-halide and its superior performance for low grade energy recovery**

**Guoliang An**†**, Liwei Wang**†*, **Jiao Gao and Ruzhu Wang**

Institute of Refrigeration and Cryogenics, Key Laboratory of Power Machinery and Engineering of MOE, Shanghai Jiao Tong University, Shanghai, 200240, China.

* Correspondence to: [lwwang@sjtu.edu.cn](mailto:lwwang@sjtu.edu.cn).

†These authors contributed equally to this work.

**Supplementary Information**

**Experimental results with test unit with PDM under equilibrium conditions**

In order to verify the experimental results of equilibrium conditions, the equilibrium experiments is performed by test unit with PDM (see Figure S4b for schematic diagram of test unit). In the experiments the matched mass among halides does not need to be considered because no resorption process occurs, thus, the mass proportion is chosen as 1:1:1 for comparison, which is same with the proportion of the study in reference^1^. The similar conclusion with experiments tested by Rubotherm balance, i.e. the resorption between different sorbents doesn’t happen, has been proved through series of equilibrium experiments (see Figure S5 for results of isobaric sorption/desorption curves and hysteresis phenomenon using test unit with PDM).

However, some special characteristics in Figure S5 need to be paid attention. First, although the reaction stage of various halides can also be distinguished, inside each one of them, the reaction is no more mono-variant controlled process, especially for sorption direction. The second aspect is that as large mass of halide sorbent is filled inside the sorption bed, some of the sorbent cannot react thoroughly and it makes the cycle sorption quantity less than that tested by Rubotherm balance. For instance, the cycle sorption quantity of CaCl_2_ tested by Rubotherm balance is 5.96 mol$\cdot$mol^-1^, while that value is just 4.75 mol$\cdot$mol^-1^ for test unit with PDM.

**Statistical analysis of data**

For Rubotherm balance test unit, the sorption quantity and cycle sorption quantity are defined as:

$x=\left(m_{\text{a}}-m_{0}\right)/m_{\text{s}}$ (S1)

$\Delta x=\left(m_{\text{a}}-m_{\text{d}}\right)/m_{\text{s}}$ (S2)

where $m_{\text{a}}$ and $m_{0}$ are the mass of basket during testing processes and at the initial state without sorption respectively, $m_{\text{s}}$ is the mass of sorbent, $m_{\text{d}}$ is the mass of basket at the completed desorption state. The uncertainty of the sorption quantity and cycle sorption quantity are defined as:

$\varepsilon^{*}\left(x\right)=\frac{\varepsilon\left(m_{\text{s}}\right)}{m_{\text{s}}}+\frac{\varepsilon\left(m_{\text{a}}\right)+\varepsilon\left(m_{0}\right)}{m_{\text{a}}-m_{0}}$ (S3)

$\varepsilon^{*}\left(\Delta x\right)=\frac{\varepsilon\left(m_{\text{s}}\right)}{m_{\text{s}}}+\frac{\varepsilon\left(m_{\text{a}}\right)+\varepsilon\left(m_{\text{d}}\right)}{m_{\text{a}}-m_{\text{d}}}$ (S4)

where $\varepsilon^{*}$ is the maximum fractional error, $\varepsilon$ is the maximum absolute error (20 μg for $\varepsilon\left(m\right)$). As long as the calculated sorption quantity or cycle sorption quantity is larger than 0.01 g∙g^-1^, the uncertainty is less than 1%.

For test unit with PDM, the cycle sorption quantity is defined as:

$\Delta x=\frac{1}{m_{\text{s}}}\cdot\left\{\left[1-\frac{v'(T_{e})}{v"(T_{e})}\right]\cdot\frac{A_{\text{e}}}{g}\cdot\Delta(\Delta p)\right\}$ (S5)

where $m_{\text{s}}$ is the sorbent mass (g), $v'(T_{e})$and $v"(T_{e})$ are specific volume of saturated liquid and saturated vapour ammonia (m^3^∙kg^-1^), $A_{\text{e}}$ is the cross-section area of refrigerant in the evaporator/condenser (mm^2^), g is the gravity acceleration (9.80 m∙s^-2^), $\Delta p$ is the pressure difference between the vapour and liquid ends of evaporator/condenser (Pa), $\Delta(\Delta p)$ is the variation of the pressure difference (Pa). Because the ratio between $v"(T_{e})$ and $v'(T_{e})$ is almost 1,000, that part is neglected during uncertainty analysis. The uncertainty of the cycle sorption quantity is defined as:

$\varepsilon^{*}\left(\Delta x\right)=\frac{\varepsilon\left(m_{\text{s}}\right)}{m_{\text{s}}}+\frac{\varepsilon\left(A_{\text{e}}\right)}{A_{\text{e}}}+\frac{\left(\Delta p_{1}\right)\times\varepsilon^{*}\left(\Delta p_{1}\right)+\left(\Delta p_{2}\right)\times\varepsilon^{*}\left(\Delta p_{2}\right)}{\left|\Delta p_{1}-\Delta p_{2}\right|}$ (S6)

where $\varepsilon\left(m_{\text{s}}\right)$ is 0.01 g, $\varepsilon\left(A_{\text{e}}\right)$ is 1.51 mm^2^, $\varepsilon^{*}\left(\Delta p_{1}\right)$ and $\varepsilon^{*}\left(\Delta p_{2}\right)$ are 0.65%. According to the definition of *COP* (equation (7)) and $\eta_{\text{e}}$ (equation (8)), the uncertainty of them both equal to twice of $\varepsilon^{*}\left(\Delta x\right)$. The calculated uncertainty results are shown in Figure S7 and S8.

The reaction enthalpy and entropy are obtained by least square fitting as shown in equation (S7):

$\ln p=-\frac{\Delta H}{R}\times\frac{1}{T}+\frac{\Delta S}{R}$ (S7)

where $p$ is the pressure of evaporator/condenser, $\Delta H$and $\Delta S$ are the reaction enthalpy and entropy when complexing a molecular unit of ammonia, *R* is the universal gas constant, and *T* represents the reaction temperature in the reactor. The uncertainty of the calculated $\Delta H$ and $\Delta S$ are defined as equation (S8, S9):

$\varepsilon^{*}(\Delta H)=\frac{\left[\Delta S\text{+}R\times\text{ln}\left(p\right)\right]\times p\times\varepsilon\left(T\right)+R\times T\times p\times\varepsilon^{*}\left(p\right)}{T\times p\times\left[\Delta S\text{-}R\times\ln\left(p\right)\right]}+\sqrt{\frac{1-R^{2}}{\left(n-2\right)\times R^{2}}}$ (S8)

$\varepsilon^{*}(\Delta S)=\frac{p\times\Delta H\times\varepsilon\left(T\right)+R\times T^{2}\times p\times\varepsilon^{*}\left(p\right)}{T\times p\times\left[\Delta H+R\times T\times\ln\left(p\right)\right]}+\frac{\Delta H}{\Delta S}\sqrt{\frac{1-R^{2}}{\left(n-2\right)\times R^{2}}\times\overline{\left(\frac{1}{T^{2}}\right)}}$ (S9)

where $\varepsilon\left(T\right)$ is 0.1 K, $\varepsilon^{*}\left(p\right)$ is 0.04%, *n* is the number of calculation points (equals to 4 for CaCl_2_ and 6 for MnCl_2_). Results of calculated enthalpy and entropy are shown in Table S1.

**SI Figure Legends**

**Figure S1.** Single-stage sorption cycle. (a) Clapeyron diagram. (b) Desorption phase with MnCl_2_ as example. (c) Sorption phase with MnCl_2_ as example.

**Figure S2.** Preparation procedures of composite sorbents. (a) Sorbent used in the Rubothermal balance test unit. (b) Sorbent used in test unit with PDM.

**Figure S3.** Pre-treatment results of sorbents using Rubotherm balance test unit. (a) NH_4_Cl under the condition of vacuum and heating temperature of 60^o^C. **(**b) Multi halide sorbent under the condition of vacuum and heating temperature of 180^o^C.

**Figure S4.** Schematic diagram of test unit for sorption quantity measurement. (a) Rubotherm balance test unit. (b) Test unit with PDM.

**Figure S5.** Isobaric sorption/desorption curves of multi-halide sorbent (1:1:1) and CaCl_2_ under equilibrium conditions. (a), (c) For multi-halide sorbent, the sorption quantity is the sorbed mass of ammonia by multi-halide sorbent over the mass of multi-halide sorbent itself, at 0.441 MPa (a) and 0.622 MPa (c) respectively. (b), (d) For the CaCl_2_ inside multi-halide sorbent, the sorption quantity is the sorbed mass of ammonia by CaCl_2_ over the mass of CaCl_2_ itself, at 0.441 MPa (b) and 0.622 MPa (d) respectively.

**Figure S6.** Sorption quantity vs time under non-equilibrium conditions, related to Fig. 4. (a) At 0.441 MPa. (b) At 0.622 MPa.

**Figure S7.** Uncertainty of sorption quantity, related to the results of Fig. 4. (a) At 0.441 MPa. (b) At 0.622 MPa.

**Figure S8.** Uncertainty of *COP* and $\eta_{\text{e}}$, related to the results of Fig. 6b.

**SI Table Legend**

**Table S1.** Reaction enthalpy and entropy considering the influence of hysteresis.

**Supplementary data**

The initial data of Fig. 2 include files whose names are: Fig.2b-CaCl_2_-0^o^C, Fig.2b-CaCl_2_-10^o^C-1, Fig.2b-CaCl_2_-10^o^C-2, Fig.2b-CaCl_2_-10^o^C -3, Fig.2b-CaCl_2_-20^o^C, Fig.2b-CaCl_2_-30^o^C, and Fig.2c-MnCl_2_.

The initial data of Fig. 3 include files whose names are: Fig.3-Multi-0^o^C, Fig.3-Multi-10^o^C, Fig.3-Multi-20^o^C, and Fig.3-Multi-30^o^C.

The initial data of Fig. 4 include files whose names are: Fig.4-CaCl_2_-0-10^o^C, Fig.4-Multi-0^o^C, and Fig.4-Multi-10^o^C.

The initial data of Fig. 6 include files whose names are: Fig.6-CaCl_2_-0^o^C, Fig.6-CaCl_2_-20^o^C, Fig.6-Multi-0^o^C, and Fig.6-Multi-20^o^C.

The initial data of Supplementary Fig. 5 include files whose names are: SFig.5-CaCl_2_-0^o^C-1, SFig.5-CaCl_2_-0^o^C-2, SFig.5-CaCl_2_-10^o^C, SFig.5-Multi-0^o^C, and SFig.5-Multi-10^o^C.

**Supplementary References**

1 Gao, J., Wang, L. W., Wang, R. Z. & Zhou, Z. S. Solution to the sorption hysteresis by novel compact composite multi-salt sorbents. *Applied Thermal Engineering* **111**, 580-585 (2017).


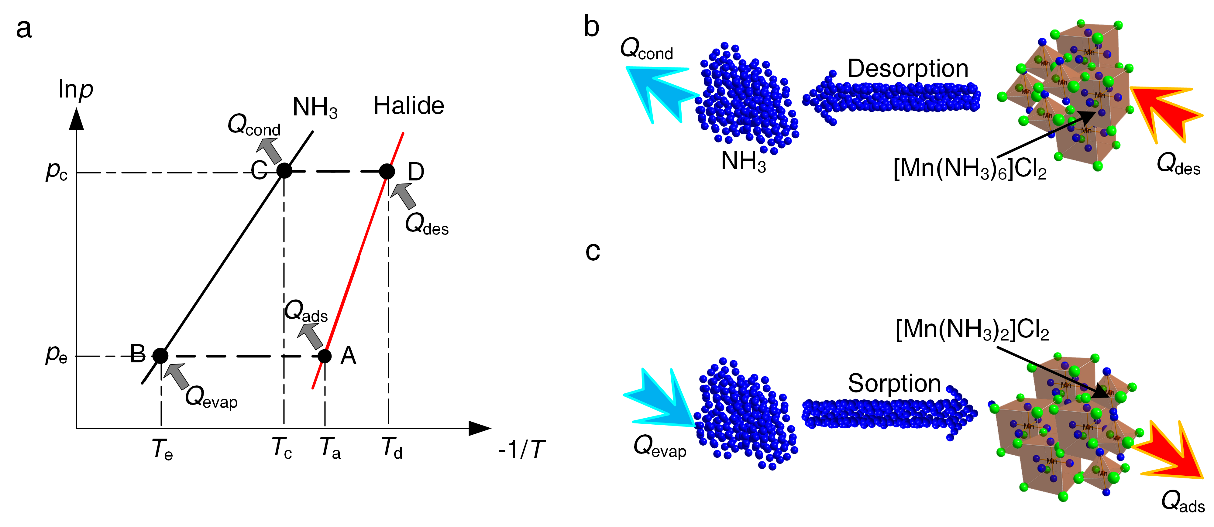


**Figure S1**


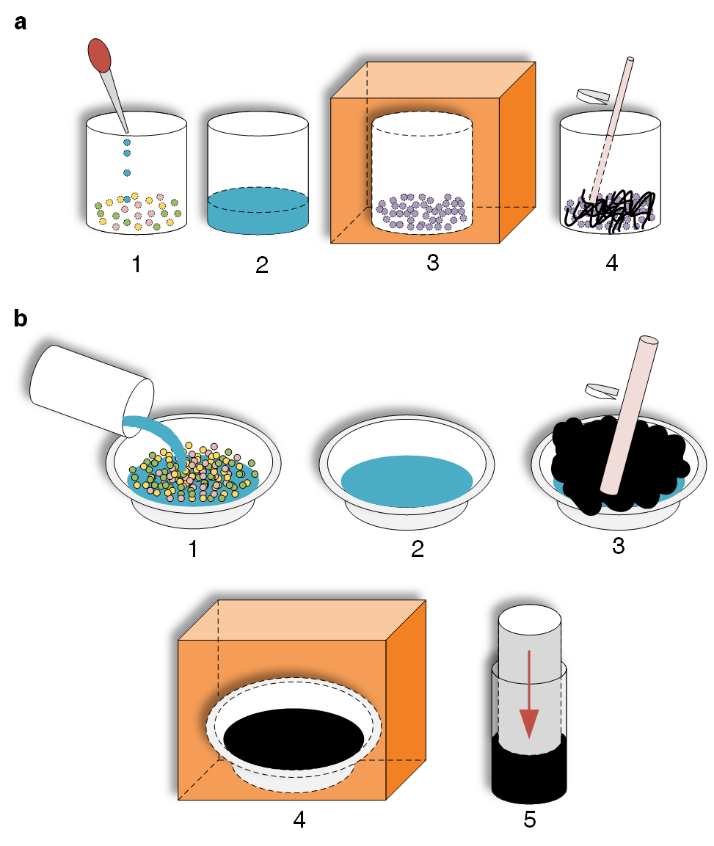


**Figure S2**


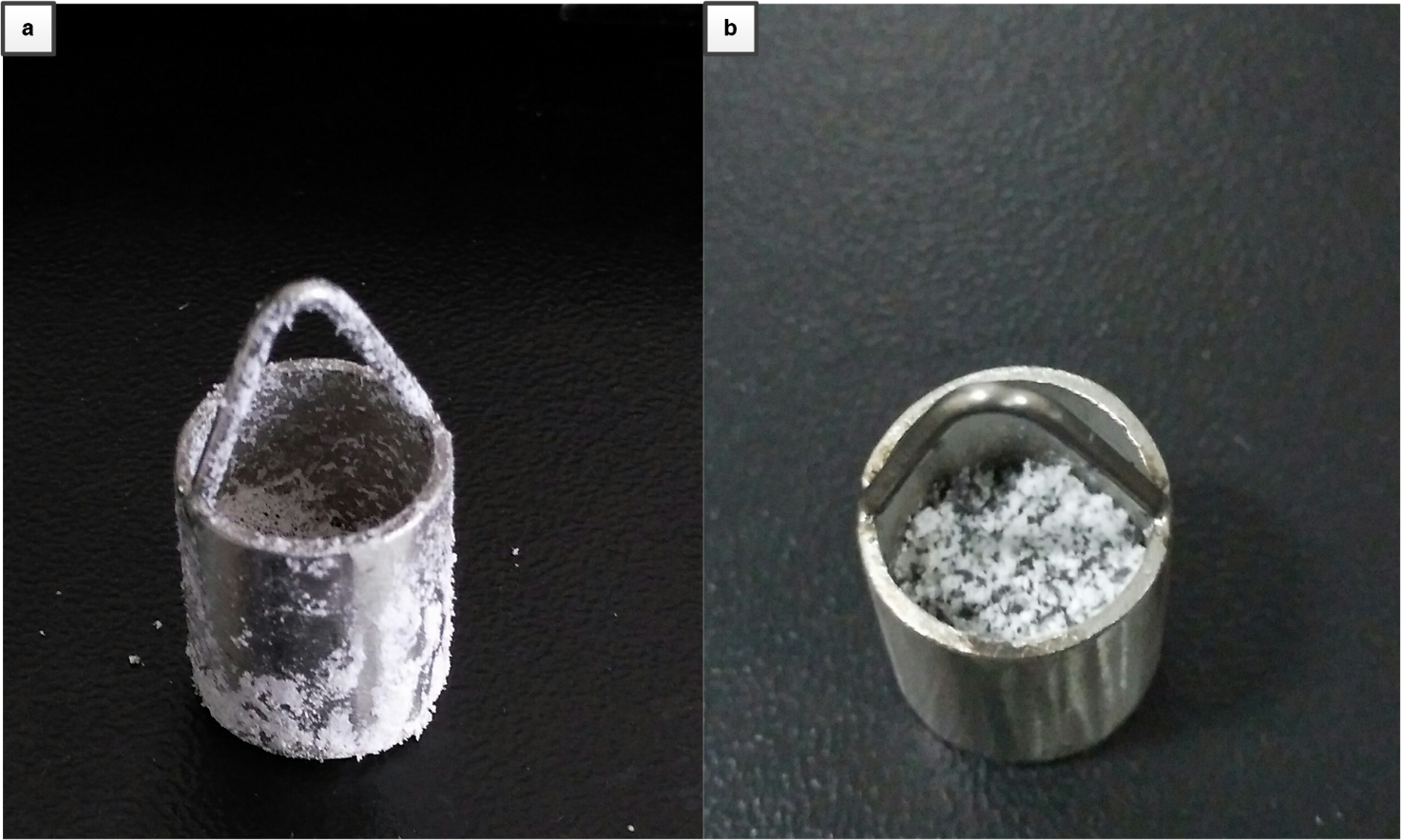


**Figure S3**


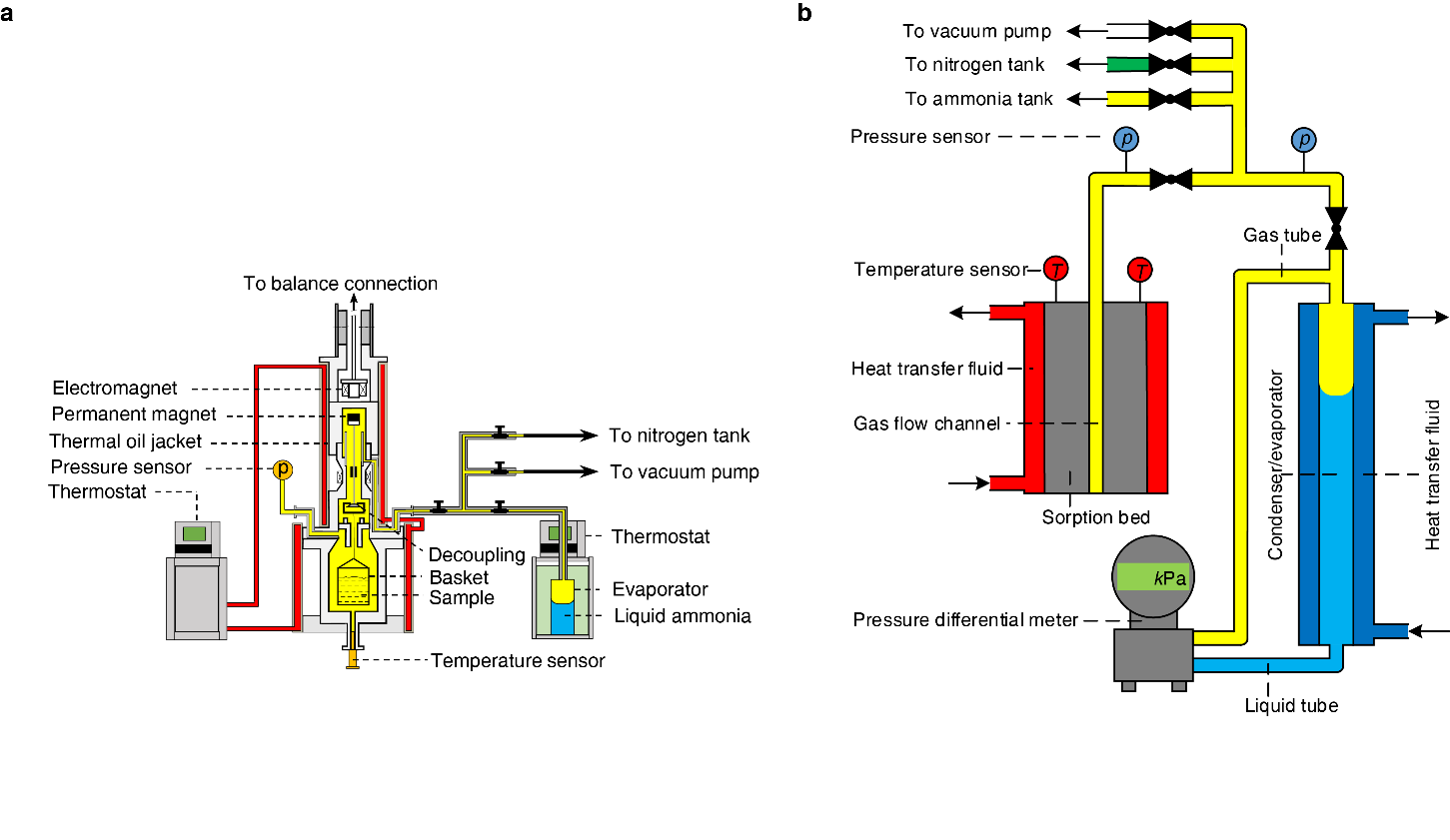


**Figure S4**


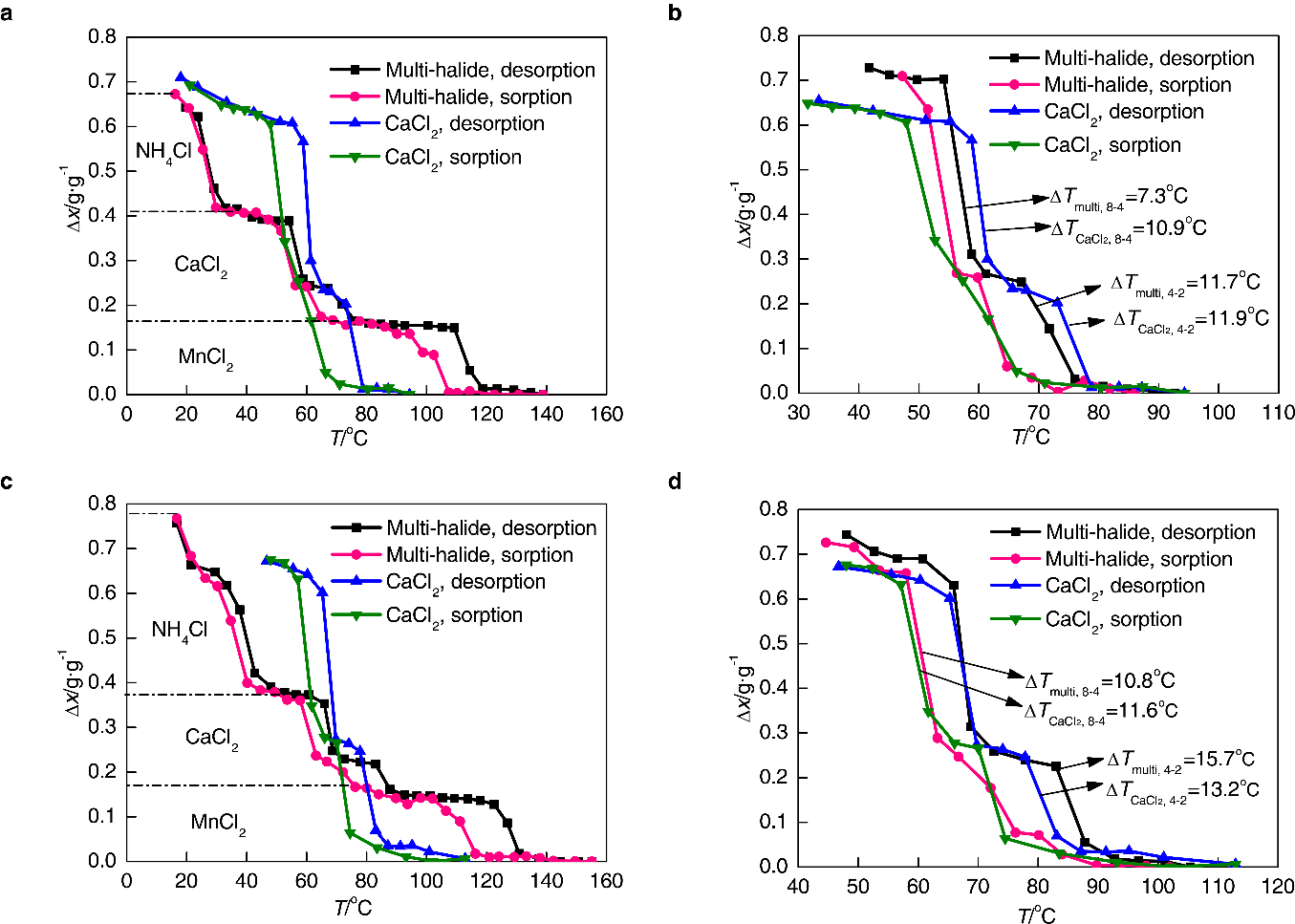


**Figure S5**

**
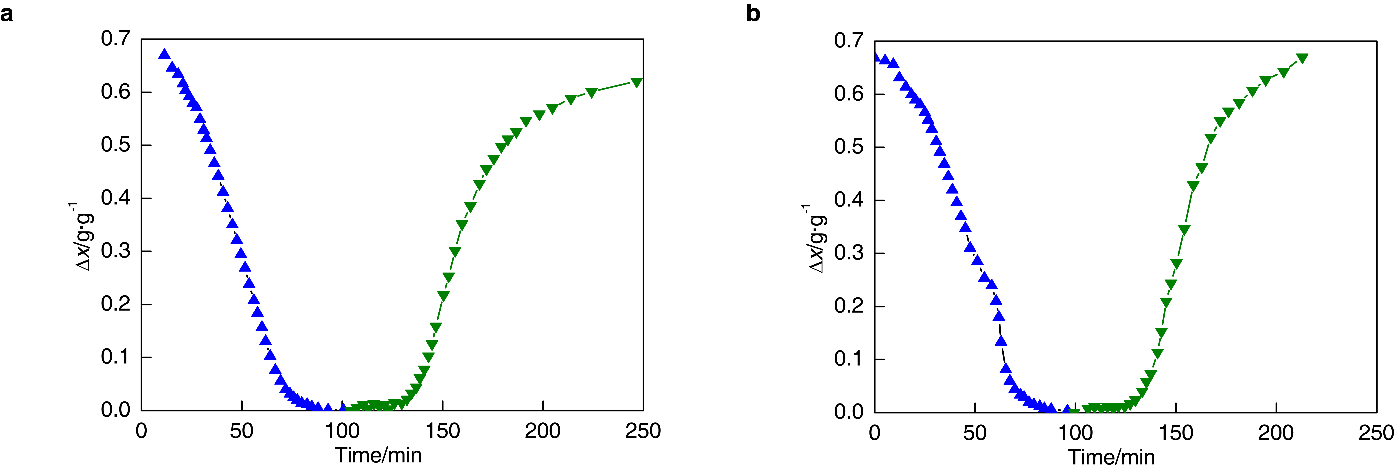
**

**Figure S6**


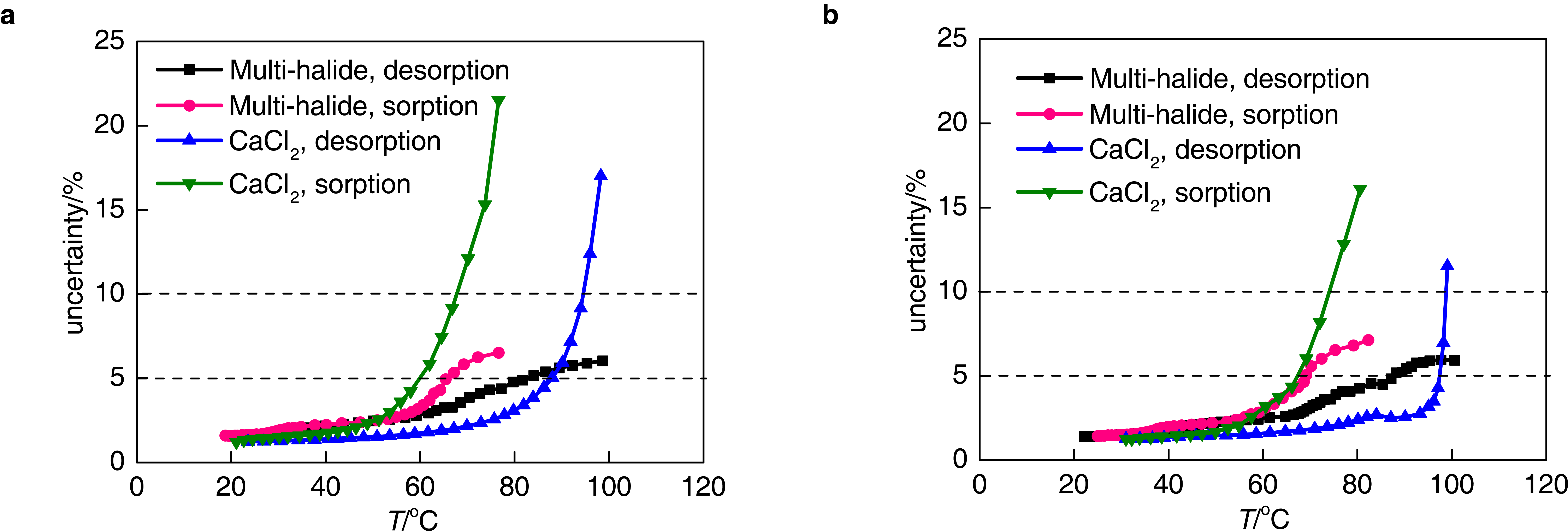


**Figure S7**


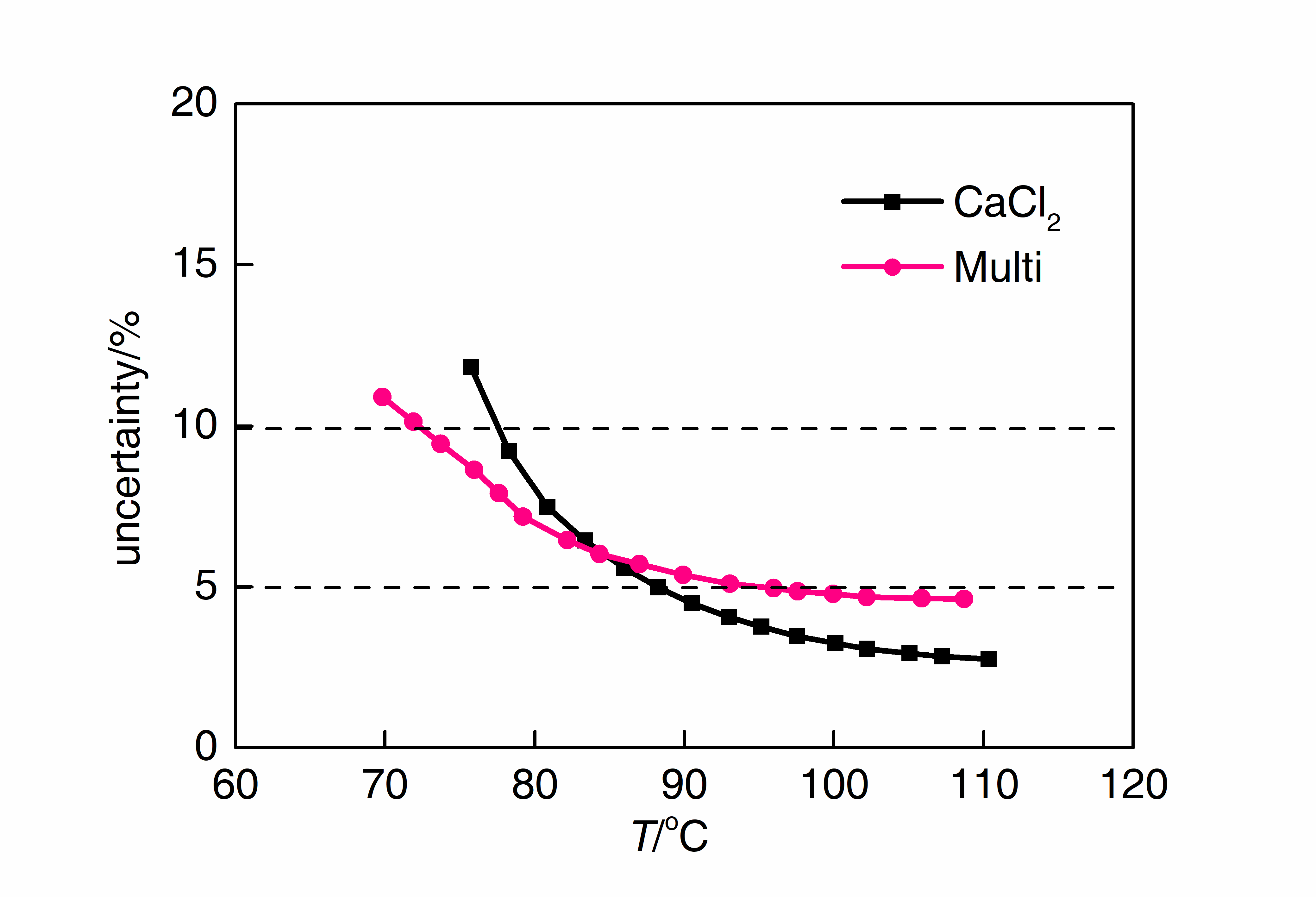


**Figure S8**

**Table S1**

| Reaction | ∆*H* (J$\cdot$mol^-1^) | $\varepsilon^{*}(\Delta H)$ | ∆*S* (J$\cdot$mol^-1^$\cdot$K^-1^) | $\varepsilon^{*}(\Delta S)$ | R^2^ |
| --- | --- | --- | --- | --- | --- |
| Ca 4-8 | 43549 | 4.37% | 241.85 | 2.35% | 0.9962 |
| Ca 8-4 | 40732 | 8.73% | 229.37 | 4.29% | 0.9850 |
| Ca 2-4 | 41119 | 7.03% | 231.38 | 3.63% | 0.9902 |
| Ca 4-2 | 45420 | 13.70% | 235.95 | 7.20% | 0.9638 |
| Mn 2-6 | 41273 | 1.32% | 214.95 | 0.65% | 0.9993 |
| Mn 6-2 | 52221 | 1.23% | 238.62 | 0.67% | 0.9994 |
